# Measurement of the volume CT dose index on spiral CT scanning with a real‐time ionization chamber

**DOI:** 10.1002/acm2.70469

**Published:** 2026-01-26

**Authors:** Atsushi Fukuda, Nao Ichikawa, Takuma Hayashi, Ayaka Hirosawa, Kosuke Matsubara

**Affiliations:** ^1^ Department of Radiological Sciences, School of Health Sciences Fukushima Medical University Fukushima Fukushima Japan; ^2^ Department of Radiological Technology, Faculty of Health Science Kobe Tokiwa University Kobe Hyogo Japan; ^3^ Department of Radiology Shiga Psychiatric Medical Center Kusatsu Shiga Japan; ^4^ Department of Medical Technology Toyama Prefectural Central Hospital Toyama Toyama Japan; ^5^ Department of Quantum Medical Technology, Faculty of Health Sciences, Institute of Medical, Pharmaceutical and Health Sciences Kanazawa University Kanazawa Ishikawa Japan

**Keywords:** direct measurement, radiation dose rate profile, spiral scanning, volume computed tomography dose index

## Abstract

**Background:**

The measurement of computed tomography dose index 100 ($CTDI_{100}$), which is feasible only through axial scanning, requires that the clinical spiral protocols be replaced with those for axial scanning. The real‐time ionization chamber detects the integral of radiation dose rate profile, enabling the direct verification of the volume CTDI on spiral CT scanning ($CTDI_{vol}^{Spiral}$).

**Purpose:**

This study aimed to develop a direct measurement technique for $CTDI_{vol}^{Spiral}$ and compare its accuracy with that measured using axial scanning ($CTDI_{vol}^{Axial}$) or that displayed on the console ($CTDI_{vol}^{Displayed}$).

**Methods:**

A CTDI phantom with a real‐time ionization chamber was placed on the headrest or examination table. $CTDI_{100}^{Axial}$ was measured with following parameters: tube voltage = 120 kV, effective mAs = 100, and rotation time = 1.00 s. The parameters for measuring $CTDI_{100}^{Spiral}$ were set identical to those used for axial scanning, except for rotation times = 0.33, 0.50, and 1.00, pitch = 0.35, 0.50, 0.75, 1.00, 1.25, and 1.50, and the scanning range = 15 cm. $CTDI_{100}^{Spiral}$ was extracted from the integral of radiation dose rate profile. $CTDI_{vol}^{Spiral}$ was subsequently calculated and compared with $CTDI_{vol}^{Axial}$ and $CTDI_{vol}^{Displayed}$. Finally, $CTDI_{vol}^{Spiral}$ was measured for 10 clinical protocols and compared with $CTDI_{vol}^{Displayed}$.

**Results:**

The differences between $CTDI_{vol}^{Axial}$ and $CTDI_{vol}^{Displayed}$, $CTDI_{vol}^{Spiral}$ and $CTDI_{vol}^{Displayed}$, and $CTDI_{vol}^{Axial}$ and $CTDI_{vol}^{Spiral}$ for the head and body phantoms were all < −2.0%. The differences between $CTDI_{vol}^{Displayed}$ and $CTDI_{vol}^{Spiral}$ for scans on clinical protocols with and without automatic exposure control were < 11.9% and < 10.5%, respectively; these large differences were observed in the dual‐energy twin‐beam protocol. Excluding this protocol yielded differences between the measurements with and without automatic exposure control of < 2.0% and < −3.9%, respectively.

**Conclusions:**

The results showed an excellent agreement between $CTDI_{vol}^{Axial}$ and $CTDI_{vol}^{Spiral}$, supporting the use of the clinical spiral CT scanning to verify $CTDI_{vol}^{Displayed}$ using a real‐time ionization chamber.

## 1 INTRODUCTION

The United Nations Scientific Committee on the Effects of Atomic Radiation 2020/2021 report states that computed tomography (CT) accounts for only 10% of all procedures but contributes to 62% of the collective effective dose.^1^ Managing the radiation dose in CT scanning is essential to conform to the as low as reasonably achievable (ALARA) principle.^2^ Although the volume CT dose index ($CTDI_{vol}$) has been employed for radiation output monitoring for several decades,^3^ several potential limitations have been reported. First, $CTDI_{vol}$ underestimates the values when wide‐beam CT scanning is performed.^4^ Second, $CTDI_{vol}$ does not theoretically apply to perfusion studies or stational acquisitions.^5^ To overcome these limitations, the American Association of Physicists in Medicine Task Group 111 introduced the concept of equilibrium dose, reporting on the comprehensive methodology for the radiation dose evaluation in x‐ray CT.^6^ Although the equilibrium dose might serve as a new CT dose index in the near future, $CTDI_{vol}$ is currently widely accepted for radiation output monitoring in CT systems^3^ and is a mandatory requirement for the American College of Radiology CT accreditation.^7^

$CTDI_{vol}$ is defined as follows:

(1) $$\;CTDI_{vol}=\frac{1}{Pitch}\;\left(\frac{1}{3}CTDI_{100,c}+\frac{2}{3}CTDI_{100,p}\right),$$

where, *pitch* is the nominal table feed in each gantry rotation (*I*) divided by the total thickness of all simultaneously acquired slices (*nT*); $CTDI_{100,c}$ and $CTDI_{100,p}$ are the average $CTDI_{100}$ values measured with a 100‐mm‐long ionization chamber located at the central axis and the four peripheral holes (1 cm depth) of the CTDI phantom, respectively. $CTDI_{100}$ is also a measurement of the radiation output integrated over 100‐mm length for a single axial scanning:

(2) $$\;CTDI_{100}=\frac{1}{nT}\;\int_{-50}^{50}D\left(z\right)dz,$$

where, D(z) is the radiation dose profile along the *z*‐axis in the 100‐mm‐long pencil ionization chamber. $CTDI_{100}$ can be measured only through axial scanning.^7^ Therefore, the clinical spiral protocols require replacing with those for axial scanning. However, this process can be time‐consuming, error‐prone, and in some cases, not possible.^8^, ^9^, ^10^

A real‐time radiation detector enables the measurement of the radiation dose rate profile ($\dot{D}\left(t\right)$), which could display the relationship between the radiation dose rate ($\dot{D}$) and time (*t*). The real‐time radiation detector is used to measure the rotation time,^11^, ^12^ table feed speed,^12^ gantry overrun,^13^, ^14^ half‐value layer,^15^, ^16^ bowtie filter,^17^ and angular tube current modulation in the CT system.^18^ When the CTDI phantom, which is embedded with the 100‐mm long real‐time ionization chamber, is spirally scanned, the ionization chamber detects the integral of the radiation dose rate profile along the *z*‐axis as a function of time $\dot{D}\left(z,t\right)$ in relation to the x‐ray tube angle and CTDI phantom (examination table) position. Therefore, the integral of $\dot{D}\left(z,t\right)$ might be momentarily proportional to the $CTDI_{100}$ when passing through the center of the CTDI phantom. Hence, we hypothesized that $CTDI_{vol}^{Spiral}$ could be directly measured through the integral of $\dot{D}\left(z,t\right)$ using the real‐time ionization chamber. This study aimed to develop a new direct measurement technique for $CTDI_{vol}^{Spiral}$ and compare its accuracy with that measured on axial scanning ($CTDI_{vol}^{Axial}$) or that displayed on the console ($CTDI_{vol}^{Display}$).

## 2 METHODS

### 2.1 Devices

A 128‐slice whole‐body CT system (SOMATOM go.Top, Siemens Healthineers, Erlangen, Germany) was employed in this study. The CT scanner featured the following settings: tube voltage of 70–140 kV; tube currents of 13–625 mA; rotation times of 0.33, 0.50, and 1.00 s; and nominal pitch factors ranging from 0.35 to 1.50 in increments of 0.05. The *nT* was 38.4 mm (64 × 0.60 mm), while CARE Dose4D and X‐CARE were utilized for automatic exposure control. The manufacturer pre‐installed the recommended clinical protocols in the CT system utilized for verifying $CTDI_{vol}^{Spiral}$ in this study.

The real‐time ionization chamber (10 × 6‐3CT, Radcal, Monrovia, CA) was employed to measure the $CTDI_{100}^{Axial}$ and the integral of $\dot{D}\left(z,t\right)$ on spiral scanning to compute $CTDI_{100}^{Spiral}$. The 100‐mm long ionization chamber with a volume of 3 cc was connected to a laptop through a digitizer and USB cable. The dedicated software (Accu‐Gold 2, Radcal, Monrovia, CA) displays the integral of $\dot{D}\left(z,t\right)$ that could export the raw data. The exported temporal resolution was 0.1024 ms. The ionization chamber was calibrated in RQR9 x‐ray beam quality.

The head (16 cm diameter) and adult body (32 cm diameter) CTDI phantoms (model 468‐BH, Sun Nuclear, Middleton, WI) were used in this study. The CTDI phantom comprises a two‐piece telescopic configuration. Each module allows the measurement of $CTDI_{100}$ on the central axis of the phantom or at the periphery positions located every 90° at 1.0 cm depth from the surface.

### 2.2 Measurement technique for determining $CTDI_{100}^{Spiral}$ from the integral of $\dot{D}\left(z,t\right)$

**FIGURE 1:**
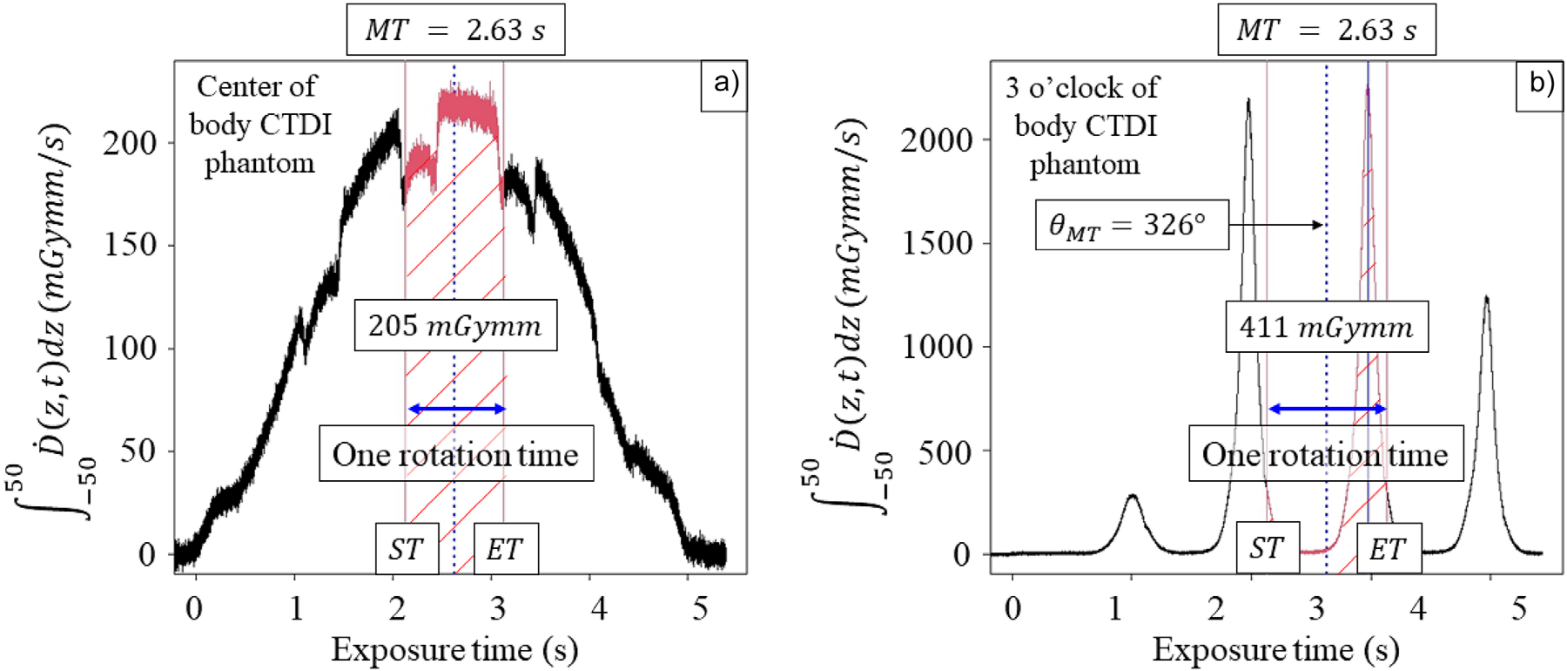
The left graph (a) shows the $\int_{-50}^{50}\dot{D}\left(z,t\right)dz$ measured on the spiral scanning for the body CTDI measurement when the real‐time ionization chamber was located at the central axis of the CTDI phantom. Although the integral of $\dot{D}\left(z,t\right)$ periodically decreased due to the table attenuation, the peak of the integral of $\dot{D}\left(z,t\right)$ was observed at the middle time (MT) of the x‐ray exposure. The start time (ST) and end time (ET) were set at the $MT\mp \frac{gantry\;rotation\;time}{2},\;\mathrm{respectively}$. $CTDI_{100}^{Spiral}$ was calculated from the time integral of $\int_{-50}^{50}\dot{D}\left(z,t\right)dz$ between ST and ET (the area marked with diagonal lines). The right graph (b) shows the $\int_{-50}^{50}\dot{D}\left(z,t\right)dz$ measured at the 3 o'clock of the CTDI phantom. The $\int_{-50}^{50}\dot{D}\left(z,t\right)dz$ profile has the periodical peaks when the x‐ray tube passes by the real‐time ionization chamber. The x‐ray tube angle at MT ($\theta_{MT}$) can be calculated using the time difference between the MT and the peak time.

The 100‐mm long real‐time ionization chamber enables the measurement of the integral of $\dot{D}\left(z,t\right)$. Then, the rate meter reading $\dot{M}\left(t\right)$ denotes as follows:^19^

(3) $$\dot{M}\;\left(t\right)=\frac{1}{100\cdot C}\;\int_{-50}^{50}\dot{D}\left(z,t\right)dz,$$

where C is the chamber calibration factor. Combining the Formulae (2) and (3), it can be seen that:

(4) $$\begin{array}{cc} nT\cdot \;CTDI_{100} & =\int_{-50}^{50}D\left(z\right)dz=\int_{ST}^{ET}\int_{-50}^{50}\dot{D}\left(z,t\right)dzdt\\ & =100\cdot C\int_{ST}^{ET}\dot{M}\left(t\right)dt \end{array}$$

where ST and ET were the start time and end time to time integrate $\dot{M}\left(t\right)$, respectively. Figure 1a displays the sample of the integral of $\dot{D}\left(z,t\right)$ measured on the spiral scanning when the real‐time ionization chamber was located at the center of the body CTDI phantom. Although the integral of $\dot{D}\left(z,t\right)$ periodically decreased due to the table attenuation, the peak of the integral of $\dot{D}\left(z,t\right)$ was observed at the middle time (MT) of the x‐ray exposure. In other words, the central x‐ray beam axis just passed through the center of the CTDI phantom. Because the exposure time was displayed on the console, the MT could be simply determined as half the exposure time. The ST and ET were set at $MT\mp \frac{rotation\;time}{2}$, respectively. Therefore, $CTDI_{100,c}^{Spiral}$ could be calculated using the Formula (4).

Figure 1b displays the sample of the integral of $\dot{D}\left(z,t\right)$ measured on the spiral scanning when the real‐time ionization chamber was located at the peripheral three o'clock of the body CTDI phantom. Similarly, $CTDI_{100,p}^{Spiral}$ could be also calculated using the Formula (4). Furthermore, the integral of $\dot{D}\left(z,t\right)$ profile has the periodical peaks when the x‐ray tube passes by the 100‐mm long real‐time ionization chamber. Because the integral of $\dot{D}\left(z,t\right)$ profile data at the peripheral location includes the peak signal between ST and ET, the x‐ray tube angle at MT ($\theta_{MT}$) can be calculated using the time difference between the MT and the peak time (PT). Therefore, when the ionization chamber is placed at the location of $\varphi_{IC}$ of CTDI phantom, $\theta_{MT}$ could be calculated as follows:

(5) $$\;\theta_{MT}=\varphi_{IC}+\left(MT-PT\right)\times \frac{360}{ET-ST},$$

If $\theta_{MT}$ is negative or greater than 360°, the true $\theta_{MT}$ is calculated by adding or subtracting 360°, respectively.

### 2.3 Measurement accuracy of $CTDI_{vol}^{Axial}$ and $CTDI_{vol}^{Spiral}$

**FIGURE 2:**
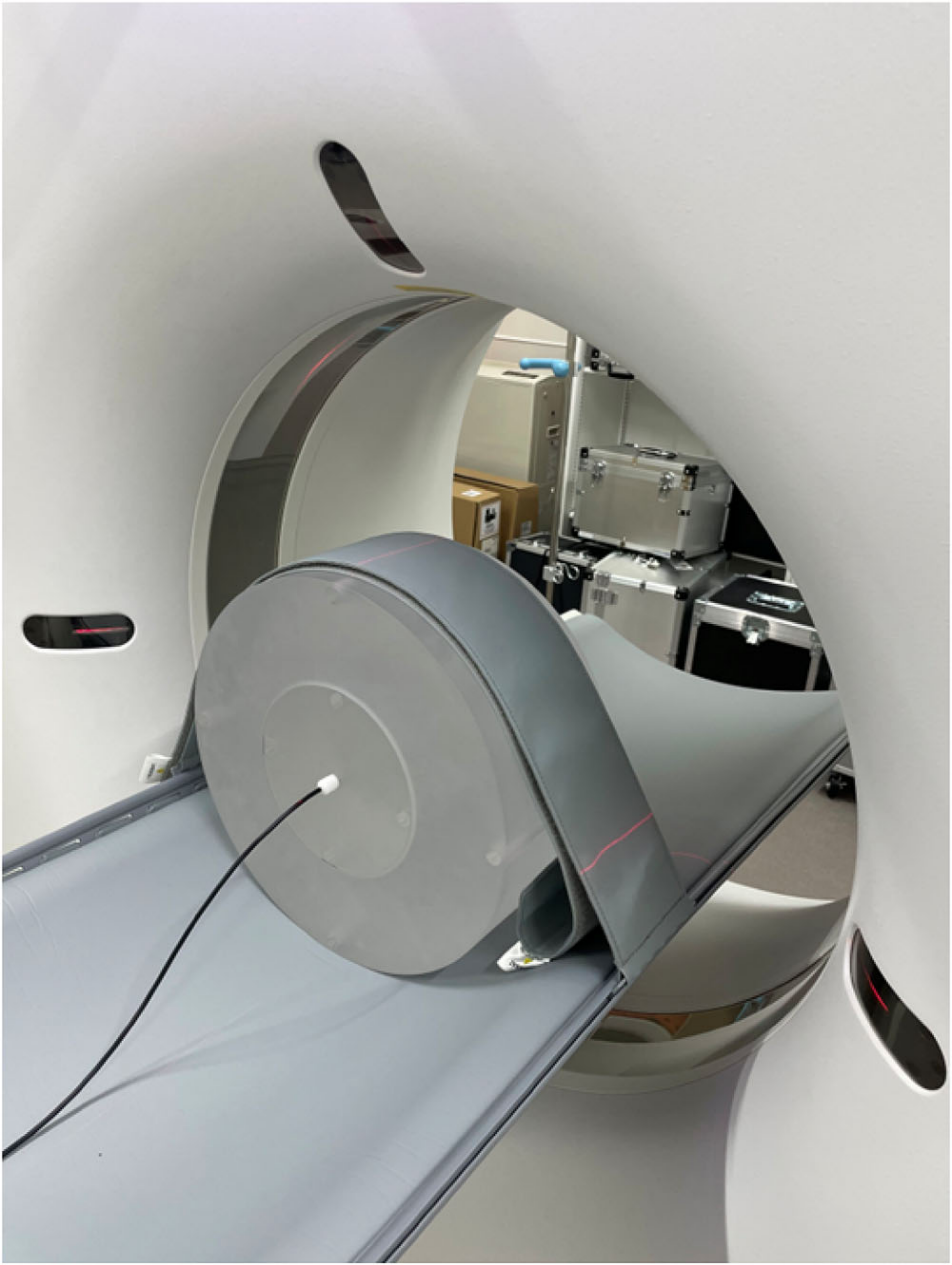
Measurement setup for the body $CTDI_{vol}$.

Figure 2 shows the measurement setup for $CTDI_{100}$ (body). The CTDI phantom was placed on the headrest or examination table for the head and body protocols, respectively. The real‐time ionization chamber was inserted on the central axis or at the four periphery positions of the CTDI phantom. The CTDI phantom position was carefully set up with a positioning laser, and the four peripheral positions were aligned to the 12, 3, 6, and 9 o'clock directions. Table 1 shows the x‐ray CT parameters for comparing the accuracy between $CTDI_{vol}^{Axial}$ and $CTDI_{vol}^{Spiral}$. The identical parameters between axial and spiral scanning were programmed, including the tube voltage, effective mAs, rotation time, collimation, and bowtie filter. The pitch factor for spiral scanning was set at 1.00. Automatic exposure control was inactive during both axial and spiral scanning.

**TABLE 1:** X‐ray CT parameters for comparing the accuracy of $CTDI_{vol}^{Axial}$ and $CTDI_{vol}^{Spiral}$.

		Scanning parameter displayed on the console									
Scanning mode	Phantom	Tube voltage (kV)	Effective mAs (mAs)	Rotation time (s)	Pitch	Collimation (mm)	Exposure time (s)	Scan direction	CARE Dose4D (CARE kV IQ level)	$CTDI_{vol}^{Displayed}$ (mGy)	DLP (mGy × cm)
Axial scanning for head CTDI measurement	Head	120	100	1.00	NA	64 × 0.6	1.01	NA	OFF (NA)	18.8 (16 cm)	72.1
Spiral scanning for head CTDI measurement					1.00		5.27	Head to Feet	OFF (NA)	18.7 (16 cm)	315
Axial scanning for body CTDI measurement	Body	120	100	1.00	NA	64 × 0.6	1.01	NA	OFF (NA)	8.98 (32 cm)	34.5
Spiral scanning for body CTDI measurement					1.00		5.26	Head to Feet	OFF (NA)	8.92 (32 cm)	151

$CTDI_{vol}$, volume computed tomography dose index; $CTDI_{vol}^{Axial}$, $CTDI_{vol}$ measured on axial scanning, $CTDI_{vol}^{Spiral}$, $CTDI_{vol}$ measured on spiral scanning, DLP, dose length product; NA, not applicant.

**FIGURE 3:**
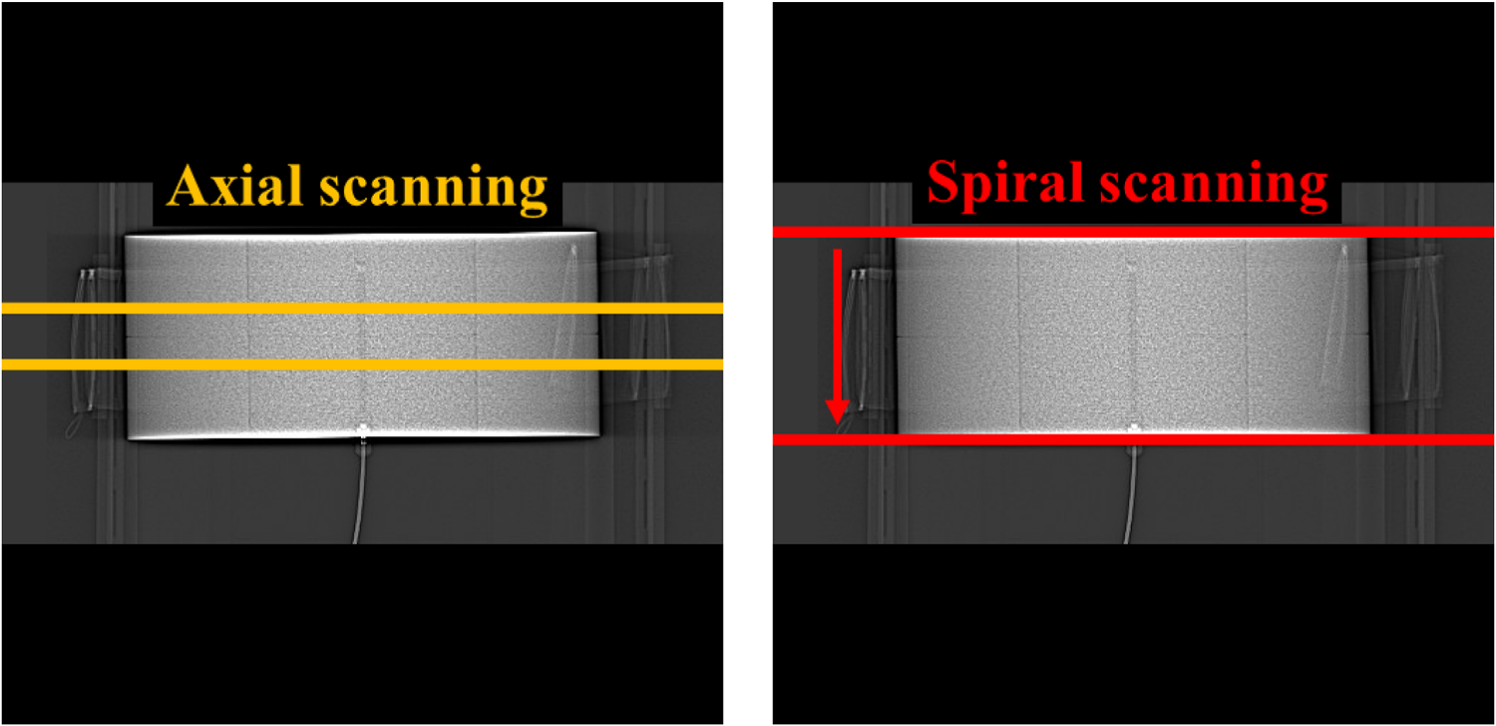
Scanning areas on axial (left) and spiral scanning (right) on the topogram.

Figure 3 depicts the area of the axial and spiral CT scanning on the topogram. The axial scanning was planned on the center of the CTDI phantom, while the spiral CT scanning was performed from the top to bottom of the CTDI phantom, with a scanning range of 15 cm. During the axial scanning, $CTDI_{100,c}^{Axial}$ and $CTDI_{100,p}^{Axial}$ were measured four times each at the center and peripheral positions of the CTDI phantom. During the spiral scanning, $\dot{M}\left(t\right)$ was acquired four times each at the center and peripheral positions of the CTDI phantom. Upon data acquisition completion, $CTDI_{100,c}^{Spiral}$ and $CTDI_{100,p}^{Spiral}$ were calculated using the Formula (4) to determine $CTDI_{vol}^{Spiral}$. The means and standard deviations of all $CTDI_{100,c}^{Axial}$, $CTDI_{100,p}^{Axial}$, $CTDI_{vol}^{Axial}$, $CTDI_{100,c}^{Spiral}$, $CTDI_{100,p}^{Spiral}$, and $CTDI_{vol}^{Spiral}$ were subsequently calculated. The $CTDI_{100,c}^{Axial}$ and $CTDI_{100,c}^{Spiral}$ as well as $CTDI_{100,p}^{Axial}$ and $CTDI_{100,p}^{Spiral}$ were compared, and finally, the $CTDI_{vol}^{Axial}$, $CTDI_{vol}^{Spiral}$, and $CTDI_{vol}^{Displayed}$ were compared with each other.

Furthermore, to investigate the effect of $\theta_{MT}$, $CTDI_{100,p}^{Spiral}$ at 12 o'clock and the $\theta_{MT}$ were measured 10 times using the Formulae (4) and (5). $CTDI_{100,p}^{Spiral}$ were subsequently plotted as a function of the $\theta_{MT}$. Moreover, to investigate the effects of the rotation time and pitch factor, the scan parameters were changed as follows: rotation times of 0.33, 0.50, and 1.00 s and pitch factors of 0.35, 0.50, 0.75, 1.00, 1.25, and 1.50. The body CTDI phantom was employed for the additional testing because the integral of $\dot{D}\left(z,t\right)$ profile shows a steeper curve in the periphery positions. Upon data acquisition completion, a Bland‐Altman analysis was performed. Subsequently, the errors were calculated as $\left(CTDI_{vol}^{Spiral}-CTDI_{vol}^{Displayed}\right)/CTDI_{vol}^{Displayed}$, and plotted as a function of the pitch factor.

### 2.4 Comparison of $CTDI_{vol}^{Displayed}$ and $CTDI_{vol}^{Spiral}$ on clinical protocols

The geometrical setting and the scanning area were identical to those mentioned in Section 2.3. Table 2 shows the clinical x‐ray CT parameters for comparing $CTDI_{vol}^{Displayed}$ with $CTDI_{vol}^{Spiral}$. The clinical protocols consist of the spiral scanning from the head to the pelvis. Nine of the 10 protocols were selected for adult patients, while the remaining one was for child patients. Two protocols for the head and the abdomen were dual‐energy twin‐beam scanning, wherein the x‐ray beam was split into two energy spectra using gold (Au) and tin (Sn) filters. All scanning parameters were pre‐installed in the CT system. No changes were made, except for the automatic exposure control (CARE Dose4D and X‐CARE). The nine clinical protocols were programmed with CARE Dose4D, while the head (child) scan was programmed with X‐CARE. The automatic exposure control was set as active or inactive for comparing the $CTDI_{vol}^{Displayed}$ and $CTDI_{vol}^{Spiral}$.

**TABLE 2:** Clinical x‐ray CT parameters employed in this study.

Scanning mode		Phantom	Tube voltage (kV)	Effective mAs (mAs)	Rotation time (s)	Pitch	Collimation (mm)	Exposure time (s)	Scan direction	CARE Dose4D (CARE kV IQ level)	$CTDI_{vol}^{Displayed}$ (mGy)	DLP (mGy × cm)
1	Head (adult) spiral scanning	Head	120	231	1.00	0.55	64 × 0.60	8.35	Feet to Head	ON (345) OFF (NA)	43.4 (16 cm) 43.2 (16 cm)	683 675
2	Head CTA (adult) Spiral scanning	Head	120	232	0.50	0.55	64 × 0.60	4.20	Feet to Head	ON (345) OFF (NA)	43.4 (16 cm) 43.5 (16 cm)	684 681
3	Head dual energy twin beam	Head	AuSn 120	697	0.50	0.25	64 × 0.60	9.62	Feet to Head	ON (345) OFF (NA)	38.3 (16 cm) 38.2 (16 cm)	708 707
4	Head (Child) Spiral scanning	Head	100	314	1.00	0.55	64 × 0.60	8.34	Feet to Head	X‐CARE OFF (NA)	36.7 (16 cm) 37.0 (16 cm)	575 578
5	Neck (adult) spiral scanning	Body	120	179	1.00	0.80	64 × 0.60	6.17	Head to Feet	ON (156) OFF (NA)	16.0 (32 cm) 16.0 (32 cm)	263 260
6	Thorax (adult) spiral scanning	Body	120	78	0.33	1.2	64 × 0.60	1.47	Head to Feet	ON (78) OFF (NA)	6.95 (32 cm) 6.98 (32 cm)	122 119
7	Abdomen (adult) spiral scanning	Body	120	119	0.50	0.80	64 × 0.60	3.10	Head to Feet	ON (143) OFF (NA)	10.7 (32 cm) 10.6 (32 cm)	178 174
8	Abdomen CTA (adult) spiral scanning	Body	120	120	0.50	0.80	64 × 0.60	3.08	Head to Feet	ON (143) OFF (NA)	10.7 (32 cm) 10.7 (32 cm)	176 173
9	Abdomen dual energy twin beam	Body	AuSn 120	328	0.33	0.30	64 × 0.60	5.23	Head to Feet	ON (143) OFF (NA)	9.11 (32 cm) 9.11 (32 cm)	166 166
10	Pelvis (adult) spiral scanning	Body	120	104	1.00	0.80	64 × 0.60	6.21	Head to Feet	ON (143) OFF (NA)	9.25 (32 cm) 9.27 (32 cm)	155 151

$CTDI_{vol}$, volume computed tomography dose index; DLP, dose length product; NA, not applicant.

$\dot{M}\left(t\right)$ was acquired four times each at the center and peripheral positions of the CTDI phantom. Upon data acquisition completion, $CTDI_{vol}^{Spiral}$ was determined using the $CTDI_{100,c}^{Spiral}$ and $CTDI_{100,p}^{Spiral}$ calculated using the Formula (4). The means and standard deviations of $CTDI_{100,c}^{Spiral}$, $CTDI_{100,p}^{Spiral}$, and $CTDI_{vol}^{Spiral}$ were calculated, and the $CTDI_{vol}^{Spiral}$ was compared with the $CTDI_{vol}^{Displayed}$. Furthermore, a Bland‐Altman analysis was also performed. Subsequently, $CTDI_{vol}^{Displayed}$ was plotted as a function of the $CTDI_{vol}^{Spiral}.$

## 3 RESULTS

Figure 1a shows the integral of $\dot{D}\left(z,t\right)$ measured on the spiral scanning as a function of the exposure time at the central axis of the body CTDI phantom. X‐ray exposure was performed at a tube voltage of 120 kV, an effective mAs of 100, a rotation time of 1.00 s, a pitch factor of 1.00, and a scanning range of 15 cm, as shown in Table 1. The exposure time of 5.26 s was displayed on the console. Therefore, the MT, ST, and ET set to 2.63, 2.13, and 3.13 s, respectively. $nT\cdot CTDI_{100,c}^{Spiral}$ was 205 mGymm, and $CTDI_{100,\;c}^{Spiral}$ was 5.34 mGy. Figure 1b also shows the integral of $\dot{D}\left(z,t\right)$ measured on the spiral scanning as a function of the exposure time at the three o'clock position of the body CTDI phantom. The MT of the exposure time was 2.63 s, and $nT\cdot CTDI_{100,p}^{Spiral}$ was 411 mGymm. Similarly, $nT\cdot CTDI_{100,p}^{Spiral}$ at the 6, 9, and 12 o'clock positions were 387, 411, and 419 mGymm, respectively. Therefore, $CTDI_{100,p}^{Spiral}$ was 10.6 mGy. Finally, $CTDI_{vol}^{Spiral}$ was determined as 8.8 ± 0.2 mGy.

Table 3 shows the accuracy and precision of $CTDI_{100,c}^{Axial}$, $CTDI_{100,p}^{Axial}$, $CTDI_{vol}^{Axial}$, $CTDI_{100,c}^{Spiral}$, $CTDI_{100,p}^{Spiral}$, and $CTDI_{vol}^{Spiral}$. The differences between $CTDI_{100,\;c}^{Axial}$ and $CTDI_{100,\;c}^{Spiral}$ as well as $CTDI_{100,\;p}^{Axial}$ and $CTDI_{100,\;p}^{Spiral}$ for the head and body phantoms were both < −2.3%. Similarly, the differences between $CTDI_{vol}^{Displayed}$ (axial) and $CTDI_{vol}^{Axial}$, $CTDI_{vol}^{Displayed}$ (spiral) and $CTDI_{vol}^{Spiral}$, and $CTDI_{vol}^{Axial}$ and $CTDI_{vol}^{Spiral}$ for the head and body phantoms were < 1.1%, < −0.9%, and < −2.0%, respectively. Of note, both $CTDI_{vol}^{Displayed}$ (axial) and $CTDI_{vol}^{Axial}$ were greater than $CTDI_{vol}^{Displayed}$ (spiral) and $CTDI_{vol}^{Spiral}$ because the gantry overrun affected the $CTDI_{vol}^{Displayed}$ (axial) and $CTDI_{vol}^{Axial}$.^13^

**TABLE 3:** Accuracy and precision of $CTDI_{100,c}$, $CTDI_{100,p}$ and $CTDI_{vol}$ measured on axial and spiral scanning.

	Console	Axial scanning			Spiral scanning with a real‐time ionization chamber		
Phantom	$CTDI_{vol}^{Displayed\;}$ [mGy]	$CTDI_{100,\;c}^{Axial}$ [mGy]	$CTDI_{100,\;p}^{Axial}$ [mGy]	$CTDI_{vol}^{Axial}$ [mGy] (Difference to $CTDI_{vol}^{Displayed}$ [mGy, %])	$CTDI_{100,\;c}^{Spiral}$ [mGy] (Difference to $CTDI_{100,\;c}^{Axial}$ [mGy, %])	$CTDI_{100,\;p}^{Spiral}$ [mGy] (Difference to $CTDI_{100,\;p}^{Axial}$ [mGy, %])	$CTDI_{vol}^{Spiral}$ [mGy] (Differences to $CTDI_{vol}^{Displayed}$ [mGy, %], $CTDI_{vol}^{Axial}$ [mGy, %])
Head	18.8 ± 0.0 (Axial) 18.7 ± 0.0 (Spiral)	18.4 ± 0.0	19.3 ± 0.3	19.0 ± 0.2 (0.2 ± 0.2, 1.1%)	18.0 ± 0.0 (−0.4 ± 0.0, −2.2%)	19.1 ± 0.3 (−0.2 ± 0.4, −1.1%)	18.7 ± 0.2 (0.0 ± 0.2, 0.0%) (−0.3 ± 0.3, −1.5%)
Body	8.97 ± 0.0 (Axial) 8.92 ± 0.0 (Spiral)	5.46 ± 0.01	10.8 ± 0.2	9.0 ± 0.1 (0.1 ± 0.1, 0.6%)	5.34 ± 0.00 (−0.12 ± 0.01, −2.3%)	10.6 ± 0.4 (−0.2 ± 0.4, −1.9%)	8.8 ± 0.2 (−0.1 ± 0.2, −0.9%) (−0.2 ± 0.3, −2.0%)

$CTDI_{vol}$, volume computed tomography dose index; $CTDI_{100,c}$, CTDI measured on the central axis of the CTDI phantom using 100 mm ionization chamber; $CTDI_{100,p}$, CTDI measured at the peripheral positions of the CTDI phantom using 100 mm ionization chamber.

These data shows means and standard deviations of $CTDI_{100}$ and $CTDI_{vol}$.

Differences to $CTDI_{100\;or\;vol}$ on axial scanning or displayed data are calculated as follows:.

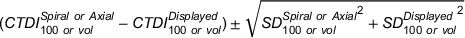
 (mGy) and $\left(CTDI_{100\;or\;vol}^{Spiral\;or\;Axial}-CTDI_{100\;or\;vol}^{Displayed}\right)/CTDI_{100\;or\;vol}^{Displayed}\times 100$ (%).

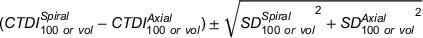
 (mGy) and $\left(CTDI_{100\;or\;vol}^{Spiral}-CTDI_{100\;or\;vol}^{Axial}\right)/CTDI_{100\;or\;vol}^{Axial}\times 100$ (%).

**FIGURE 4:**
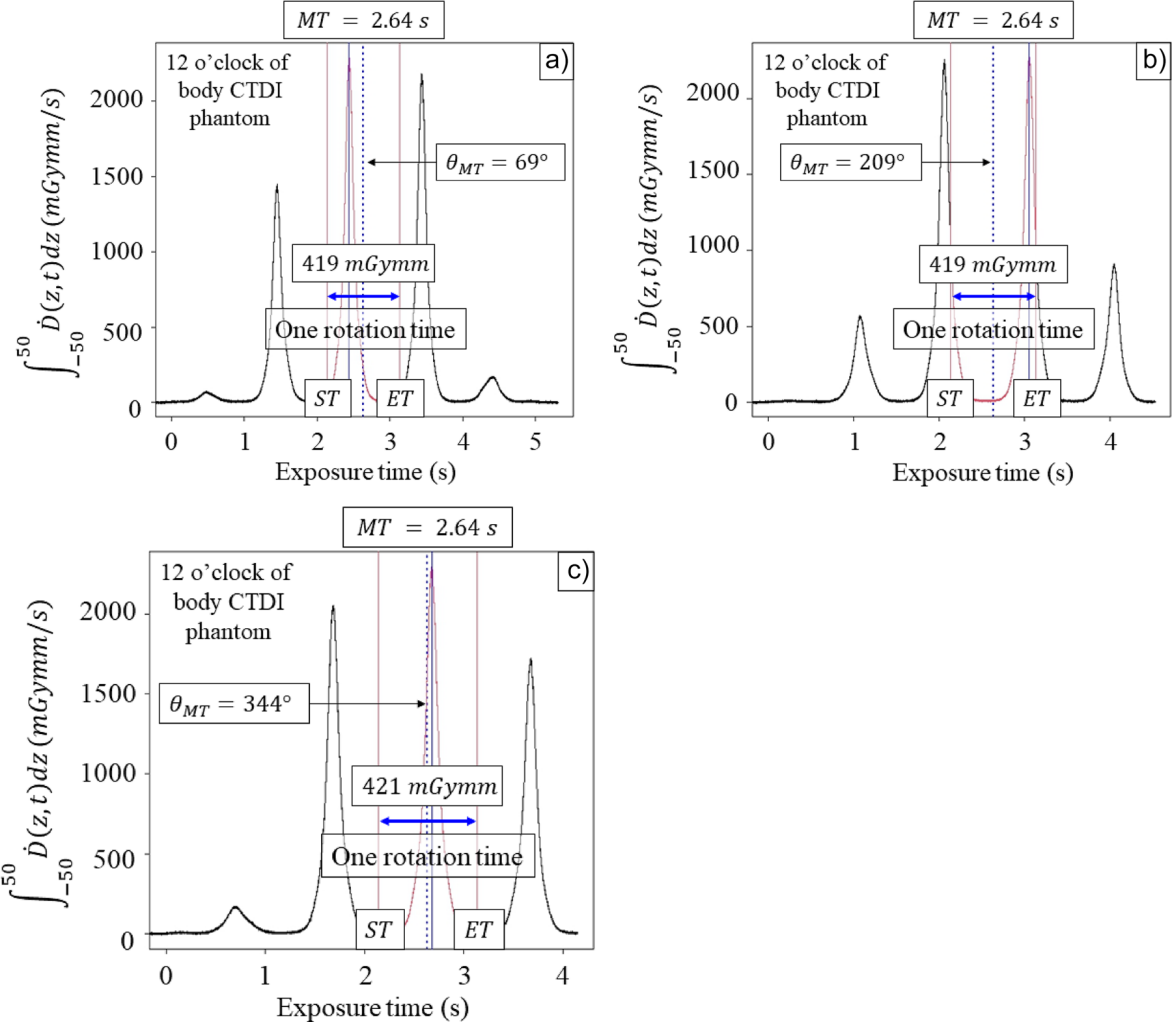
The graphs (a–c) show the three results of $\int_{-50}^{50}\dot{D}\left(z,t\right)dz$ measured at the 12 o'clock of the CTDI phantom. The x‐ray tube angle at MT ($\theta_{MT}$) differs due to the gantry starting angle.

**FIGURE 5:**
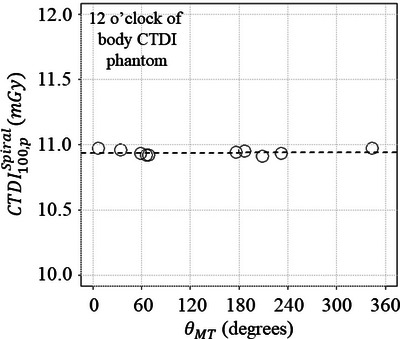
The relationship between the x‐ray tube angle at MT ($\theta_{MT}$) and $CTDI_{100,p}^{Spiral}$ measured at the 12 o'clock of the CTDI phantom.

Figure 4a–c show the samples of the integral of $\dot{D}\left(z,t\right)$ profile measured on the spiral scanning as a function of the exposure time at the 12 o'clock position of the body CTDI phantom. Because the gantry starting angle varies from scan to scan, the different $\theta_{MT}$ were observed. Figure 5 shows the $CTDI_{100,p}^{Spiral}$ as a function of the $\theta_{MT}$. These results show that the mean and standard deviation of $CTDI_{100,p}^{Spiral}$ at the 12 o'clock position of the body CTDI phantom were 10.9 ± 0.0 mGy. Because the mean and standard deviation for $CTDI_{100,p}^{Spiral}$ measured at the four peripheral locations were 10.6 ± 0.4 mGy as shown in Table 3, the uncertainty introduced by the gantry angle at MT was much less than that for four $CTDI_{100,p}^{Spiral}$. These data imply that the gantry starting angle is independent of the accuracy of the $CTDI_{100,p}^{Spiral}.$

**FIGURE 6:**
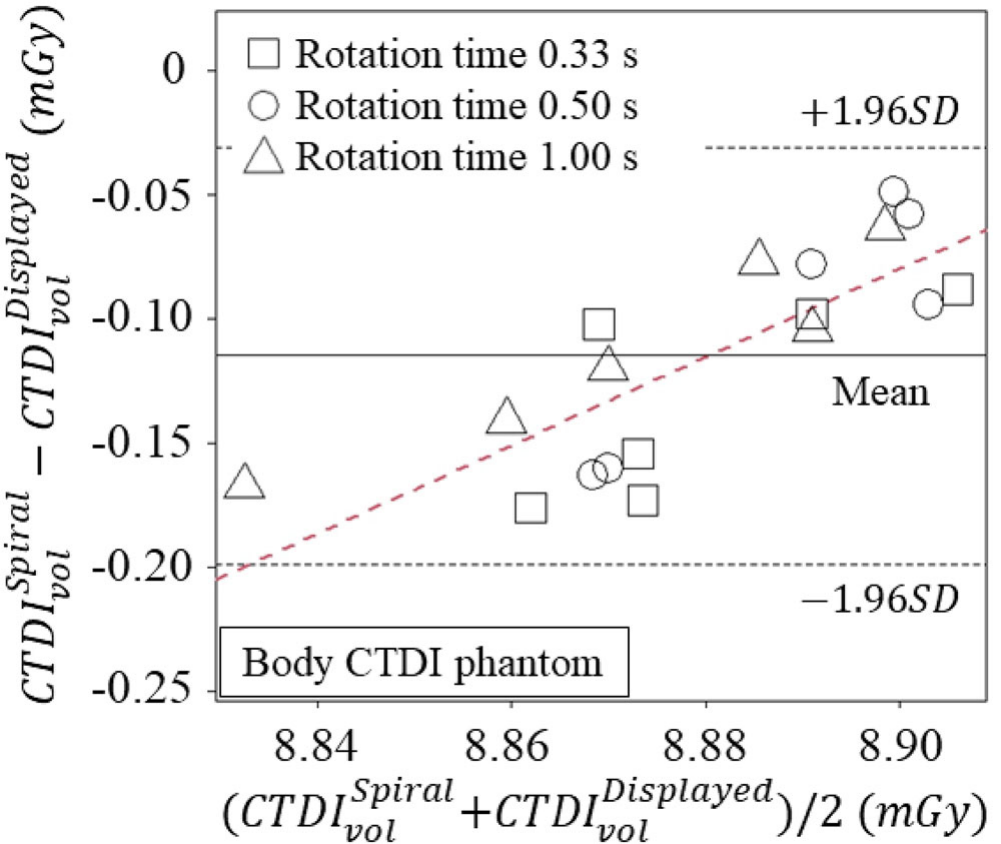
The Bland‐Altman plot between $CTDI_{vol}^{Spiral}$ and $CTDI_{vol}^{Displayed}$. These data were measured with following parameters: tube voltage = 120 kV, effective mAs = 100, and rotation times = 0.33, 0.50, and 1.00, pitch = 0.35, 0.50, 0.75, 1.00, 1.25, and 1.50, and the scanning range = 15 cm.

**FIGURE 7:**
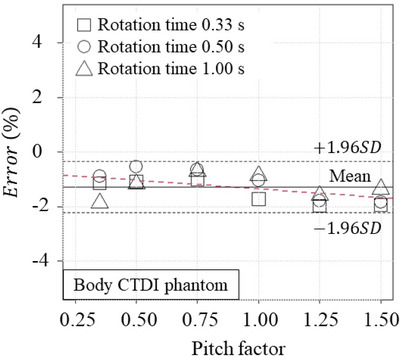
The relationship between the pitch factor and the errors of $CTDI_{vol}^{Spiral}.$ The errors were calculated as $\left(CTDI_{vol}^{Spiral}-CTDI_{vol}^{Displayed}\right)/CTDI_{vol}^{Displayed}$.

Figure 6 shows the Bland–Altman plot between the $CTDI_{vol}^{Spiral}$ and $CTDI_{vol}^{Displayed}$ when the rotation times and pitch factors were changed. The mean ± standard deviation of the differences (vertical value) was −0.11 ± 0.04 mGy. The differences were decreased with increase of the average of $CTDI_{vol}^{Spiral}$ and $CTDI_{vol}^{Displayed}$. Figure 7 shows the errors as a function of the pitch factor. The negative errors were increased with increase of the pitch factor. However, the mean ± 95% confidence interval for all errors were −1.3% ± 0.9%, and the uncertainty was much smaller than ± 20% as required by accreditation agencies.^20^

**FIGURE 8:**
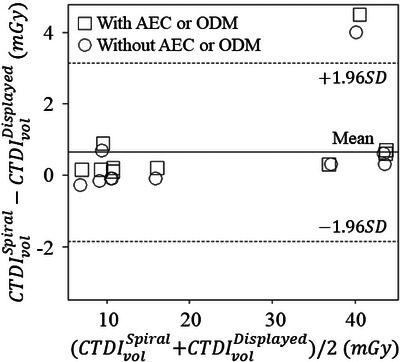
The Bland‐Altman plot between $CTDI_{vol}^{Spiral}$ and $CTDI_{vol}^{Displayed}$ measured in clinical protocols. The large differences were observed in the head dual‐energy twin‐beam protocol. AEC, automatic exposure control; ODM, organ dose modulation.

**FIGURE 9:**
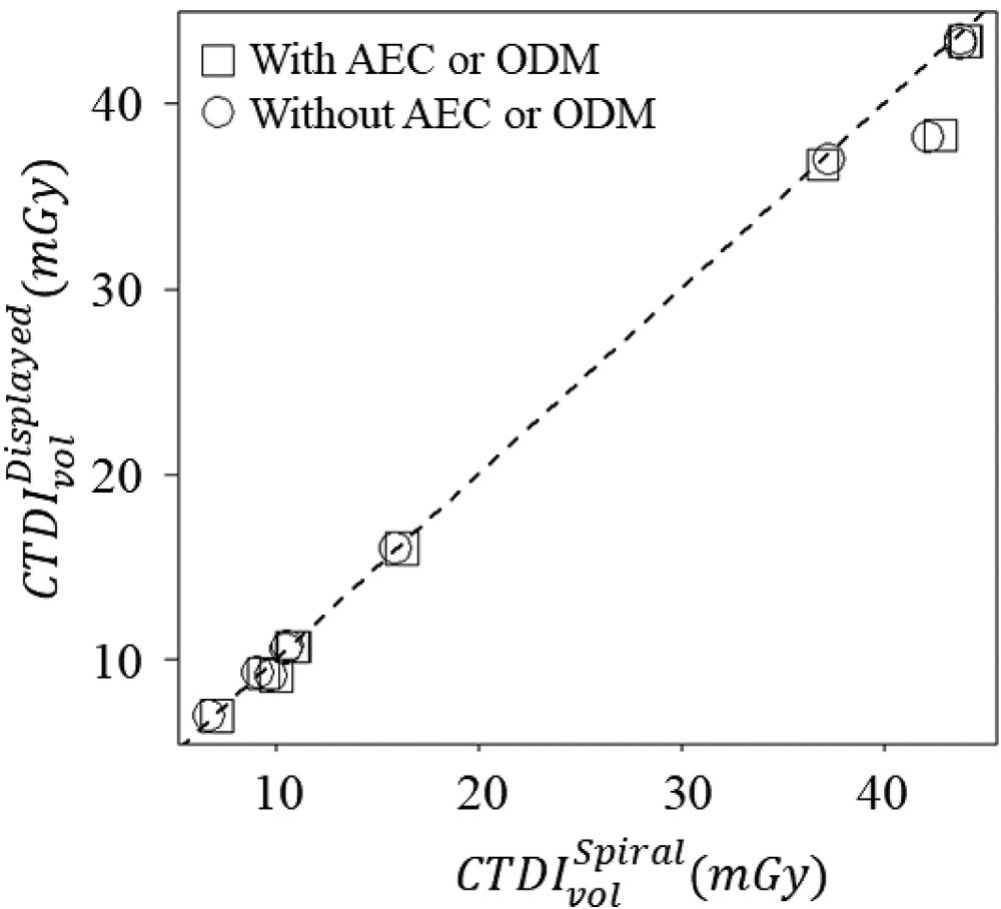
The relationship between $CTDI_{vol}^{Spiral}$ and $CTDI_{vol}^{Displayed}$ measured in clinical protocols. The large differences were observed in the head dual‐energy twin‐beam protocol. AEC, automatic exposure control; ODM, organ dose modulation.

Tables 4 and 5 show the accuracy and precision of $CTDI_{100,c}^{Spiral}$, $CTDI_{100,p}^{Spiral}$, and $CTDI_{vol}^{Spiral}$ with and without automatic exposure control. Figures 8 and 9 show the Bland‐Altman plot between the $CTDI_{vol}^{Spiral}$ and $CTDI_{vol}^{Displayed}$ and the relationship between $CTDI_{vol}^{Spiral}$ and $CTDI_{vol}^{Displayed}$ in the clinical protocols. Although the clinical protocol contains wide variations of the effective mAs, rotation time, and pitch factor, the differences between $CTDI_{vol}^{Displayed}$ and $CTDI_{vol}^{Spiral}$ with and without automatic exposure control were < 4.5 mGy (11.9%) and 4.0 mGy (10.5%), respectively. These large differences were observed in the dual‐energy twin‐beam protocol; excluding the protocol yielded differences between $CTDI_{vol}^{Displayed}$ and $CTDI_{vol}^{Spiral}$ with and without automatic exposure control of < 2.0% and < −3.9%, respectively.

**TABLE 4:** Accuracy and precision of $CTDI_{100}^{Spiral}$ and $CTDI_{vol}^{Spiral}$ measurements in clinical spiral CT scanning with automatic exposure control or organ dose modulation.

			Console	Spiral scanning with a real‐time ionization chamber		
Scanning mode		Phantom	$CTDI_{vol}^{Displayed\;}$ [mGy]	$CTDI_{100,\;c}^{Spiral}$ [mGy]	$CTDI_{100,\;p}^{Spiral}$ [mGy]	$CTDI_{vol}^{Spiral}$ [mGy] (Differences to $CTDI_{vol}^{Displayed}$ [mGy, %])
1	Head (Adult) spiral scanning	Head	43.4 ± 0.0	23.3 ± 0.0	24.6 ± 0.6	44.0 ± 0.8 (0.6 ± 0.8, 1.4%)
2	Head CTA (Adult) Spiral scanning	Head	43.4 ± 0.0	23.4 ± 0.0	24.7 ± 0.6	44.1 ± 0.8 (0.7 ± 0.8, 1.5%)
3	Head dual energy twin beam	Head	38.3 ± 0.0	10.5 ± 0.0	10.8 ± 0.2	42.8 ± 0.6 (4.5 ± 0.6, 11.9%)
4	Head (Child) Spiral scanning	Head	36.7 ± 0.1	19.6 ± 0.0	21 ± 5	37 ± 6 (1 ± 6, 1.9%)
5	Neck (Adult) spiral scanning	Body	16.0 ± 0.0	7.79 ± 0.0	15.5 ± 0.9	16.2 ± 0.8 (0.2 ± 0.8, 1.0%)
6	Thorax (Adult) spiral scanning	Body	6.95 ± 0.01	5.05 ± 0.07	10.2 ± 1.0	7.1 ± 0.6 (0.1 ± 0.6, 1.4%)
7	Abdomen (Adult) spiral scanning	Body	10.7 ± 0.00	5.23 ± 0.00	10.4 ± 1.1	10.9 ± 0.9 (0.2 ± 0.9, 1.6%)
8	Abdomen CTA (Adult) spiral scanning	Body	10.7 ± 0.00	5.25 ± 0.01	10.4 ± 1.0	10.8 ± 0.8 (0.1 ± 0.8, 1.3%)
9	Abdomen dual energy twin beam	Body	9.11 ± 0.00	1.94 ± 0.00	3.5 ± 0.3	10.0 ± 0.7 (0.9 ± 0.7, 10.2%)
10	Pelvis (Adult) spiral scanning	Body	9.25 ± 0.00	4.55 ± 0.00	9.0 ± 0.9	9.4 ± 0.8 (0.2 ± 0.8, 2.0%)

$CTDI_{vol}$, volume computed tomography dose index; $CTDI_{100,c}$, CTDI measured on the central axis of the CTDI phantom using 100 mm ionization chamber; $CTDI_{100,p}$, CTDI measured at the peripheral positions of the CTDI phantom using 100 mm ionization chamber.

These data shows means and standard deviations of $CTDI_{100}$ and $CTDI_{vol}$.

Differences to $CTDI_{vol}^{Displayed}$ is calculated as follows:.

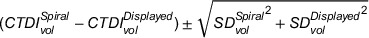
 (mGy) and $\left(CTDI_{vol}^{Spiral}-CTDI_{vol}^{Displayed}\right)/CTDI_{vol}^{Displayed}\times 100$ (%).

**TABLE 5:** Accuracy and precision of $CTDI_{100}^{Spiral}$ and $CTDI_{vol}^{Spiral}$ measurements in clinical spiral CT scanning without automatic exposure control or organ dose modulation.

			Console	Spiral scanning with a real‐time ionization chamber		
Scanning mode		Phantom	$CTDI_{vol}^{Displayed\;}$ [mGy]	$CTDI_{100,\;c}^{Spiral}$ [mGy]	$CTDI_{100,\;p}^{Spiral}$ [mGy]	$CTDI_{vol}^{Spiral}$ [mGy] (Differences to $CTDI_{vol}^{Displayed}$ [mGy, %])
1	Head (Adult) spiral scanning	Head	43.2 ± 0.0	23.2 ± 0.0	24.5 ± 0.4	43.8 ± 0.5 (0.6 ± 0.5, 1.4%)
2	Head CTA (Adult) spiral scanning	Head	43.5 ± 0.0	23.3 ± 0.0	24.5 ± 0.4	43.8 ± 0.5 (0.3 ± 0.5, 0.7%)
3	Head dual energy twin beam	Head	38.2 ± 0.0	10.4 ± 0.0	10.6 ± 0.1	42.2 ± 0.4 (4.0 ± 0.4, 10.5%)
4	Head (Child) spiral scanning	Head	37.0 ± 0.0	20.0 ± 0.0	20.8 ± 0.8	37.3 ± 0.9 (0.3 ± 0.9, 0.9%)
5	Neck (Adult) spiral scanning	Body	16.0 ± 0.0	7.67 ± 0.00	15.3 ± 0.6	15.9 ± 0.5 (−0.1 ± 0.5, −0.4%)
6	Thorax (Adult) spiral scanning	Body	6.98 ± 0.00	4.89 ± 0.00	9.6 ± 0.4	6.7 ± 0.2 (−0.3 ± 0.2, −3.9%)
7	Abdomen (Adult) spiral scanning	Body	10.6 ± 0.0	5.05 ± 0.00	10.0 ± 0.4	10.5 ± 0.3 (−0.1 ± 0.3, −1.3%)
8	Abdomen CTA (Adult) spiral scanning	Body	10.7 ± 0.00	5.10 ± 0.00	10.1 ± 0.3	10.6 ± 0.3 (−0.1 ± 0.3, −1.1%)
9	Abdomen dual energy twin beam	Body	9.11 ± 0.00	1.90 ± 0.00	3.5 ± 0.1	9.8 ± 0.3 (0.7 ± 0.3, 7.8%)
10	Pelvis (Adult) spiral scanning	Body	9.27 ± 0.02	4.41 ± 0.00	8.8 ± 0.3	9.1 ± 0.3 (−0.1 ± 0.3, −1.4%)

$CTDI_{vol}$, volume computed tomography dose index; $CTDI_{100,c}$, CTDI measured on the central axis of the CTDI phantom using 100 mm ionization chamber; $CTDI_{100,p}$, CTDI measured at the peripheral positions of the CTDI phantom using 100 mm ionization chamber.

These data shows means and standard deviations of $CTDI_{100}$ and $CTDI_{vol}$.

Differences to $CTDI_{vol}^{Displayed}$ is calculated as follows:.

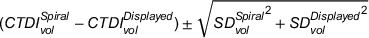
 (mGy) and $\left(CTDI_{vol}^{Spiral}-CTDI_{vol}^{Displayed}\right)/CTDI_{vol}^{Displayed}\times 100$ (%).

## 4 DISCUSSION

This study aimed to investigate the accuracy of the $CTDI_{vol}^{Spiral}$ and the feasibility of directly using clinical spiral CT scanning to verify $CTDI_{vol}^{Displayed}$ using a real‐time ionization chamber. The differences between $CTDI_{vol}^{Axial}$ and $CTDI_{vol}^{Displayed}$, $CTDI_{vol}^{Spiral}$ and $CTDI_{vol}^{Displayed}$, and $CTDI_{vol}^{Axial}$ and $CTDI_{vol}^{Spiral}$ for the head and body phantoms were < 1.1%, < −0.9%, and < −2.0%, respectively. The differences between $CTDI_{vol}^{Spiral}$ and $CTDI_{vol}^{Displayed}$ were also < 2.0% when the rotation times and pitch factors were changed. On the other hand, except for the dual‐energy twin‐beam protocols, the differences between $CTDI_{vol}^{Displayed}$ and $CTDI_{vol}^{Spiral}$ in clinical spiral CT scanning with and without automatic exposure control were < 2.0% and < −3.9%, respectively.

The International Commission on Radiation Units and Measurements report 87 introduced the application of the real‐time radiation detector to the assessment of radiation dose in CT.^21^ The radiation detectors with real‐time (> 1 kHz) readout provide capabilities in CT dosimetry that cannot be achieved using integration‐mode radiation meters. For example, the small volume real‐time radiation detectors can be translated through the x‐ray beam at isocenter, and the temporal readout gives a trace as a function of *z* in the beam. The data could be employed to analyze the radiation beam profile along *z*‐axis. Similarly, when the real‐time radiation detector is shifted from the isocenter along *x*‐ (or *y*‐) axis, the dose rate variation is observed as functions of the distance from the x‐ray focus to the radiation detector and the bowtie attenuation. The data could be also employed to analyze the bowtie filter characteristics. Many other measurement techniques have been developed, and the real‐time ionization chambers have become routine measurement devices to evaluate the CT. One of the differences between the ICRU report and this study is the volume of the real‐time ionization chamber. The ICRU report employed the small volume ionization chamber such as 0.6 cc. In contrast, a 100‐mm‐long 3 cc ionization chamber was employed in this study.

The 3‐cc real‐time ionization chamber was inserted on the central axis or at the four periphery positions of the CTDI phantom, and the spiral CT scanning was performed from the top to bottom of the CTDI phantom in this study. Beginning of the spiral CT scanning, the x‐ray beam axis enters the top edge of the phantom, and the real‐time ionization chamber could automatically start detecting the partial primary beam and the scatter intensity. The relative primary beam and scatter intensity vary as a function of the table position (or *z*‐axis).^22^ The x‐ray beam axis reaches the middle of the phantom at the MT, at which point the same primary beam and scatter intensities as in the axial scan can be measured with a real‐time ionization chamber. Therefore, when the ionization chamber is positioned on the central axis, a peak signal would be observed at MT as shown in Figure 1a. Conversely, when the ionization chamber is positioned at the four periphery positions of the CTDI phantom, a peak signal is observed depending on the gantry angle. It should be noted that the peak signal may not be observed at the MT, because the gantry starting angle is variable as shown in Figure 4a–c. Fortunately, the $CTDI_{100,p}^{Spiral}$ was independent of the $\theta_{MT}$ as shown in Figure 5.

The direct $CTDI_{vol}^{Spiral}$ measurement technique requires the time integration during the ST and ET of the integral of $\dot{D}\left(z,t\right)$. The MT could be easily determined as half the scanning time. The ST and ET were set at $MT\mp \frac{gantry\;rotation\;time}{2}$, respectively. Fukuda et al. reported that the uncertainty of the gantry rotation time is less than 1.0 ms.^11^ When the nominal gantry rotation time is set to 1.00 s, the uncertainties of ST and ET are less than 0.5 ms. Furthermore, if significant MT errors exist, the uncertainty in $CTDI_{100,c}^{Spiral}$ should be larger than the uncertainty in $CTDI_{100,p}^{Spiral}$. However, the standard deviations of the $CTDI_{100,c}^{Spiral}$ were almost zero and smaller than those of the $CTDI_{100,p}^{Spiral}$, as shown in Table 3. Therefore, the error introduced by time setting would be negligible. Furthermore, if the high pitch protocol is programmed, the x‐ray beam axis may enter far from the center of the CTDI phantom at the time of ST and ET. If the real‐time ionization chamber detects the insufficient primary beam and scatter, it might introduce the underestimation of the integral of $\dot{D}\left(z,t\right)$. In this study, the accuracy of the $CTDI_{vol}^{Spiral}$ was measured when the pitch factor was changed from 0.35 to 1.50. The high pitch factor introduced the underestimation by < ‐2.0% as shown in Figure 7; however, the uncertainty of the $CTDI_{vol}^{Spiral}$ dosimetry was almost consistent with that of the previous $CTDI_{vol}^{Axial}$.^8^, ^10^

Leon et al. reported a direct $CTDI_{vol}^{Spiral}$ technique.^8^ They introduced a new formula as following:

$$CTDI_{vol}^{Spiral}=\frac{1}{3}\;\cdot M_{Center}^{Spiral}+\frac{2}{3}\cdot M_{Peripheral}^{Spiral}$$

where $M^{Spiral}$ is the meter reading from the spiral acquisition. Thus, the $CTDI_{vol}^{Spiral}$ is independent of nT, Pitch, and the length of the ionization chamber. They also reported the differences between $CTDI_{vol}^{Axial}$ and $CTDI_{vol}^{Spiral}$ for the head and body phantoms were < 10.0%.^8^ Moreover, Barreto, et al investigated the generalizability of the spiral methodology using a web‐based platform.^10^ They analyzed the 569 data sets measured using four CT protocols on scanners from seven CT manufactures. Although the number of measurements that differed between the $CTDI_{vol}^{Displayed}$ and $CTDI_{vol}^{Spiral}$ by more than ± 20% was 22, and most $CTDI_{vol}^{Spiral}$ was within the limit of 20%.^10^, ^20^ They discussed the potential errors in measuring the $CTDI_{vol}^{Spiral}$ indicating the possibility of an incorrect acquisition parameter, including incorrect positioning of the scan prescription by participants. Yang et al. provided the visualized CT dose distribution of the head and body CTDI phantoms.^23^ The dose distribution along *z*‐axis from the spiral acquisition depends on the pitch and beam width.^23^ Although Leon's formula is based on the distribution from the spiral acquisition being approximately flat over the length of the ion chamber. Therefore, the dose variation along *z*‐axis might increase the uncertainty. Conversely, in this study, the new technique is based on the time‐ and dose‐integral to determine the $CTDI_{vol}^{Spiral}$, and the theory is straightforward and intuitive. Because one rotation measurement data passing through the central position of the CTDI phantom is only used to determine the $CTDI_{vol}^{Spiral}$ in this study, the dose distribution along *z*‐axis is less likely to affect the dosimetry. Although the data samples in this study are much smaller than those of Barreto et al., the accuracy was comparable to that of the $CTDI_{vol}^{Axial}$ data.^8^, ^10^

As shown in Table 3, the real‐time ionization chamber can be employed to measure $CTDI_{vol}^{Spiral}$; however, Equation (4) must be utilized to determine $CTDI_{100}^{Spiral}$. If determined using the measured accumulated integral of $\dot{D}\left(z,t\right)$, $CTDI_{vol}^{Spiral}$ values for the head and body phantoms are overestimated by 175.0% and 175.9%, respectively. The measured accumulated integral of $\dot{D}\left(z,t\right)$ showed the results on multiple scanning, including the values when the x‐ray beam axis did not pass through the CTDI phantom center.

In this study, $CTDI_{vol}^{Spiral}$ measured on clinical CT scanning was compared with $CTDI_{vol}^{Displayed}$. The automatic exposure control technique, including *x*–*y*–*z* modulation (Care Dose4D) and organ dose modulation (X‐CARE), is frequently employed in clinical practice. Except for the dual‐energy protocols, the accuracy of the $CTDI_{vol}^{Spiral}$ with and without automatic exposure control were within 2.0% and −3.9%, respectively, and these values were less than the criteria (20%) recommended by the accreditation agencies.^20^ Therefore, $CTDI_{vol}^{Spiral}$ could be measured with the automatic exposure control. In contrast, the differences between $CTDI_{vol}^{Displayed}$ and $CTDI_{vol}^{Spiral}$ on the dual‐energy twin‐beam protocols were greater than those measured on conventional spiral scanning. To the best of our knowledge, there is currently no established calibration beam quality for twin‐beam system that use Au and Sn split filters. In this study, the CF obtained in the RQR9 beam quality was employed to determine the integral of $\dot{D}\left(z,t\right)$. Therefore, the uncertainty associated with the dual‐energy twin‐beam protocol might be greater than that of conventional spiral scanning.

This study has two limitations. First, the measurements were performed using a clinical CT scanner with a collimation of 38.4 mm. However, many CT scanner types with various collimation sizes are released in clinical practice, and they may select the x‐ray beam width (collimation). Theoretically, the wide x‐ray beam such as collimation of 80 mm in a sophisticated CT system might underestimate the integral of $\dot{D}\left(z,t\right)$ as well as the integral of D(z). Unfortunately, because we have no access to the CT scanner with a collimation of 80 mm, no measurement verifications were carried out. However, Boone reported the $CTDI_{100}$ efficiency (ε) as $\varepsilon =\frac{CTDI_{100}}{CTDI_{\infty }}$.^4^ According to the ε data, when the collimations of 40 and 80 mm were employed at tube voltage of 120 kV, $\varepsilon (CTDI_{100,c}^{40mm})$, $\varepsilon (CTDI_{100,p}^{40mm})$, $\varepsilon (CTDI_{100,c}^{80mm})$, and $\varepsilon (CTDI_{100,p}^{80mm})$ are 62.5%, 87.5%, 60.0%, and 85.0%, respectively. Therefore, the reduction rates of $CTDI_{100,c}$ and $CTDI_{100,p}$ by changing the collimation from 40 to 80 mm, are 4% $\left(\frac{62.5\%}{60.0\%}-1\right)$ and 3% $\left(\frac{87.5\%}{85.0\%}-1\right)$, respectively. Because these reductions are much smaller than ± 20% as required by accreditation agencies,^20^ the uncertainty of $CTDI_{vol}^{Spiral}$ at 80 mm collimation might be acceptable when the low pitch is set for the measurement. Hence, $CTDI_{vol}^{Spiral}$ must be verified for other CT scanners before clinical use. Second, among the many types of real‐time ionization chambers or solid‐state detectors that are commercially available, only one type was investigated in this study; detectors that have small volume, limited temporal resolution, or short data acquisition time are not used to measure $CTDI_{vol}^{Spiral}$. Therefore, $CTDI_{vol}^{Spiral}$ must be verified with other real‐time detectors.

## 5 CONCLUSION

In this study, we have developed a direct measurement technique for $CTDI_{vol}^{Spiral}$ on spiral scanning. The results indicate that the differences in the accuracy between $CTDI_{vol}^{Axial}$ and $CTDI_{vol}^{Spiral}$ for the head and body phantoms were < −2.0%, supporting the use of clinical spiral CT scanning to verify $CTDI_{vol}^{Displayed}$ using a real‐time ionization chamber.

## AUTHOR CONTRIBUTIONS

*Conception and design of the study*: Atsushi Fukuda, Nao Ichikawa, Takuma Hayashi, Ayaka Hirosawa, and Kosuke Matsubara. *Analysis and interpretation of data*: Atsushi Fukuda, Nao Ichikawa, and Takuma Hayashi. *Drafting and final approval of the article*: Atsushi Fukuda, Nao Ichikawa, Takuma Hayashi, Ayaka Hirosawa, and Kosuke Matsubara. *Collection and assembly of data*: Atsushi Fukuda.

## CONFLICT OF INTEREST STATEMENT

Dr. Fukuda was provided with a research fund from Toyo Medic Corporation to develop the CT dosimetry technique.
